# Unraveling the rectifying role of transportation improvement on resource misallocation among manufacturing firms in China–Facts and mechanism

**DOI:** 10.1371/journal.pone.0288390

**Published:** 2023-08-17

**Authors:** Zhaohua Zhang, Zhao Zhang, Xin Wang, Derrick Robinson

**Affiliations:** 1 School of Economics, Tianjin University of Commerce, Tianjin, China; 2 College of Information and Electrical Engineering, China Agricultural University, Beijing, China; 3 Senior Researcher & Policy Analyst, Center On Policy Initiatives, San Diego, California, United States of America; Roma Tre University: Universita degli Studi Roma Tre, ITALY

## Abstract

China’s High-speed railway (HSR) network had experienced rapid expansion during 2009 to 2013, and how the HSR expansion affects China’s economy has been considerable concerned by both policymakers and researchers. Using firm-level data, this study accessed the effects of HSR on productivity distribution and resource misallocation among manufacturing firms. Incorporating difference in difference idea into a multilevel model, the results suggest significant misallocation rectifying effect of HSR at firm level. This effect is stronger for capital-intensive firms. City-specific analysis indicates that the effects of HSR on firm-level resource misallocation varies with city size, and firms grouped in small cities gain more misallocation rectification than those grouped in big and medium cities. Market potential, which is an important way through which the HSR affects efficient allocation of production resources, is found boost the marginal effect of HSR by reducing labor market segmentation and increasing agglomeration.

## 1. Introduction

As an important component of transportation systems, the HSR with an operating speed of 200 km/h and faster plays a critical role in promoting overall economic growth. Since its reform and opening-up in 1978, China has experienced rapid economic growth at an average annual rate of 15% (National Bureau of Statistics of China, 2017). To support the economic development and accommodate fast growing travel demand, the State Council approved the first mid- and long-term Railway Network Plan in 2004, unveiling the construction of the "four vertical and four horizontal" HSR network. This plan was revised in 2008 to enhance and extend the existing HSR network. With the ongoing of HSR construction projects, by the end of 2019, the total operating mileage of the HSR had reached 34,500 kilometers, accounting for 25.3 percent of China’s total railway mileage (139,800 kilometers). Upon completion, China is expected to realize the goal of zero-distance passenger transfer, seamless logistics cohesion, and integration of transportation service.

Transportation infrastructure improvement plays an important role in reshaping the spatial organization of economic activities. Compared with traditional railway, high-speed railway significantly compresses the space-time distance between cities. Since the opening of China’s first HSR–the Beijing-Tianjin intercity line–in 2008, more than10 years has passed. With China’s dual background of low resource allocation efficiency and unbalanced regional economic development, the economic effects of HSR network expansion have been of central concern to both policymakers and academics alike. Traditionally, the emphasis has been mainly on various aspects of macro-economic development, for example, GDP growth [1, 2], land use effect [3, 4] 单击或点击此处输入文字。, urban sectoral employment [5] 单击或点击此处输入文字。, and economic disparity [6] 单击或点击此处输入文字。. However, HSR impact studies carried out at aggregate levels provide little insight in the mechanisms whereby transport improvements benefit individual firms [7] 单击或点击此处输入文字。. Macroeconomic performance is determined by the behavior of micro firms. Existing studies indicated that firm-level resource misallocation would lead to a negative and sizable effect on aggregated productivity [8] 单击或点击此处输入文字。. With China’s manufacturing data, Hsieh and Klenow (2009) [9] showed that the elimination of resource misallocation among manufacturing firms would increase total factor productivity by 30%-50%. Therefore, exploring factors associated with resource misallocation across manufacturing firms would be crucial in unveiling the mechanisms whereby micro firm performance generates macroeconomic benefits in China.

Two types of misallocation were analyzed in existing works: dispersion in output-based total factor productivity (TFPQ) and dispersion in revenue-based (as opposed to output-based) total factor productivity (TFPR) [10] 单击或点击此处输入文字。. Dispersion of TFPQ indicates the survival of low productivity firms in the left tail. When low productivity firms remain in business, high productivity firms’ access to scarce resources is limited. Deviations in TFPR represent inputs are not allocated based on their marginal revenue, leading to inefficient allocation of resources. Efficient allocation of resources refers to the allocation situation that can maximize the total social output, namely Pareto optimum allocation, and it can be achieved by promoting the free flow of resources among different regions in a closed economy [11–13] 单击或点击此处输入文字。. Consequently, in an economy with poor transportation infrastructure, resources cannot flow freely which will hinder the maximization of marginal benefits and leading to resource misallocation. This study aims to investigate the impacts of HSR on firm-level resource misallocation, and explore the underlying mechanism through which HSR affects resource allocation.

HSR can affect resource allocation among firms through various channels. The New Economic Geography theory highlights that transport infrastructure improvements that reduce transport costs promote regional transfer of production resources, and emphasizes the importance of transport costs along with market size, and economies of scale in explaining inefficient allocation of resources among firms. Key to New Economic Geography models are transport costs and the way they influence market potential. Theoretical contributions in this field have advanced our understanding of how market potential can be derived from formal spatial models and that the extent of market potential is an important determinant of efficient resource allocation among firms. Market potential in the New Economic Geography refers to the number and size of the market available at low trade costs. Existing New Economic Geography models often use geodesic distances between locations when calculating market potential, but has not considered the real transport network [14, 15]. Yet, the economically relevant distance between regions is not simply the straight distance, but the cost to reach locations through the real transport network. This study takes one step forward to explore the role of real railway network change induced by HSR by shaping market potential, and the potential mechanisms through which market potential rectifies resource misallocation among firms. With the advantages of high speed and large capacity, HSR enables firms to reach distant markets faster and at lower costs, resulting in market expansions and facilitating the reallocation of inputs from less productive to more productive firms [15] 单击或点击此处输入文字。.

This study contributes to the literature on transportation improvement and firm performance by at least three aspects. First, this study provides an insight of micro-level economic impacts of transportation infrastructure improvement. Conducting an ex-post evaluation during the year 2009–2013 for prefecture level cities, we explore the relationship between HSR construction and resource misallocation across manufacturing firms in China. Secondly, we identify the plausible mechanisms through which market potential gain induced by transport improvements benefit firms. Based on the real railway network, our market potential index accounts the change of geographical scope of markets induced by HSR construction. Given the considerable investment in transportation infrastructure in China, it is crucial to reveal the underlying mechanism whereby this investment generates economic benefits. Finally, the empirical strategy that views the opening of HSR as a natural experiment and classifies firms located in cities with HSR service as treated group leads to firms located in the same city deal with similar HSR accessibility and other city-specific characteristics. To account this fact, the different-in-difference (DID) idea is incorporated into a multilevel model to investigate the impact of HSR on firm-level resource misallocation. With firms nested within cities, the multilevel model allows the inclusion of both firm-level and city-level variables as well as different types of fixed effects. The introduction of the multilevel model controlling for the hierarchical structure of firm-level data could produce more reliable estimates. Additionally, the HSR project aims to connect cities that are politically important and economically prosperous, leading to larger and richer cities are more likely to connect to the HSR network. The nonrandom assignment of HSR stations brings endogeneity issue. To address the potential endogeneity, this study uses the city’s average elevation, average gradient, and historical railway passenger capacity of 1985 as instruments for the current distribution of the HSR station.

Data used in the analysis are collected from 2009–2013 including firm-level data for Chinese manufacturing industries, national train schedule data, and city characteristics data. Our results show that the opening of HSR in a city rectifies TFPQ dispersion and TFPR dispersion by 7.3% and 8.9%, respectively, indicating a significant misallocation- rectifying effects of the HSR. To explore the heterogeneous effect of HSR on different industries, we classify our sample as capital-intensive firms and labor-intensive firms. The results show that capital-intensive firms gain more misallocation rectification. Additionally, we find that firms located in small cities experienced larger misallocation rectification due to the construction of HSR network. Mechanism analysis results suggest that market potential increase enhances the marginal impact of HSR on TFPQ and TFPR dispersions through reducing labor market segmentation and increasing agglomeration.

The paper is organized as follows: Section 2 reviews related studies and proposes research hypotheses. Section 3 introduces the methodology, and section 4 presents the data sources and variable description. Section 5 discusses the empirical results while section 6 concludes.

## 2. Literature review and research hypotheses

With the rapid expansion of China’s HSR network, its economic effects have drawn an increasing attention of the researchers. Despite a number of studies already existing, this study complements existing work by investigating the influence of transportation infrastructure on firm-level resource misallocation through its effects on market potential. Existing studies mostly explored effects of transportation infrastructure improvement at macro level, including both the direct and indirect effects. The direct impact of HSR was mainly focused on the travel time savings and accessibility improvement [16–18] 单击或点击此处输入文字。. The indirect effects of HSR are more difficult to isolate, and most study defined effects arising from agglomeration as the indirect infrastructure effects [19, 20] 单击或点击此处输入文字。. The proximity of industries and businesses contributes to cost reduction and productivity improvement by allowing firms to share a common labor pool and enjoy knowledge spillovers [21, 22] 单击或点击此处输入文字。. With the availability of micro data, recently, the focus of transportation improvement studies is shifting to micro-economic analysis. Researchers began to investigate how transportation infrastructure improvement affects specific aspect of firm performance. Since the extent of inefficient allocation of resources in a country accounts for a very large part of the total productivity loss in the economy, the relationship between transportation and firm-level resource allocation attracts interest of researchers. Inefficient resource allocation denotes a situation in which capital and labor are poorly distributed so that less productive firms receive a larger share of capital and labor than they should according to their level of productivity [23] 单击或点击此处输入文字。. Asturias et al. (2014) [24] showed that poor transportation infrastructure was an important source of resource misallocation across firms. The improvement of transportation would increase accessibility and reduce transaction costs, which facilitates efficient allocation of production resources [17] 单击或点击此处输入文字。. Barzin et al. (2018) [15] and Chen and Silva (2014) [25] indicated that changes in agglomeration economies and effective density stemming from transportation cost reductions encompass the sharing of resources across larger geographical space and more efficient matching between employers and employees. While a number of papers identified the effects of transportation investments on resource allocation, empirical studies that have tried to quantitatively assess impacts of China’s HSR on firm-level resource misallocation are still limited. The HSR greatly shortens the space-time distances between different cities. This time cost savings boost the mobility of various factors between cities, including population, information, and technology, and thus reallocate production resources [16] 单击或点击此处输入文字。. Therefore, we put forward the following hypothesis:

Hypothesis 1. The opening of HSR will decrease the misallocation of resources among manufacturing firms in China.

The concept of market potential, which is used to measure a specific geographic area’s access to markets for inputs and outputs, has received great interest in the New Economic Geography literature. Key to new economic geography models are transport costs and the way they influence market potential. Theoretically, transport improvements reduce both transportation time and costs, which increases economic opportunities for firms through providing greater market potential. Greater market potential reduces geographic labor market segmentation which can benefit firms by gaining access to a larger pool of qualified workers, and consequently raise productivity through reducing labor misallocation due to the lack of a market-based wage determination system. Furthermore, with market expansion and integration, the geographic scope at which agglomeration economies materialize can also increase. Existing studies in this field have shown empirical evidences that improvements of transport infrastructure provide greater market potential, which can influence firm-level productivity through different mechanisms. Measuring market potential by travel time through the real transport network in Spain over the last decades, Holl (2012) [7] showed that transport infrastructure investment improves accessibility to input and output markets and thus increases market potential. Hu et al. (2020) [12] found that the improvement of urban market potential brought by commuting facilitation helped reduce the regional disparity of labor wage and the spatial misallocation of labor across cities. Researchers also found that the market expansion and integration lead to agglomerated production activities [16, 22] 单击或点击此处输入文字。. With low time cost transportation, firms or production activities are more likely to locate together to enjoy positive externalities, such as technology, factor endowments and information sharing [6] 单击或点击此处输入文字。. Therefore, the above discussion leads us to propose the following hypothesis:

Hypothesis 2. Greater market potential induced by HSR can rectify resource misallocation through reducing labor market segmentation and increasing agglomeration.

The construction of HSR network can substantially shorten the space-time distance between large cities and their surrounding small cities [16] 单击或点击此处输入文字。. Existing literature indicated that this might create a “centrifugal force effect” or a “centripetal force effect”. Zheng and Kahn (2013) [26] showed that HSR increased the possibility that residents to live in small neighboring cities and work in the core cities, which promotes the transfer of a portion of production resources from large cities to small cities. However, other researchers argued that second-tier and small cities benefit the most from HSR, while the impact on megacities could be marginal or even negative [27] 单击或点击此处输入文字。. Therefore, when analyzing the effect of HSR on firm-level resource misallocation, we believe that city size matters. Besides city size, existing work also found effects of HSR on resource allocation have firm type heterogeneity [5, 28] 单击或点击此处输入文字。. Using China’s HSR network project as a source of exogeneous variation in commuting facilitation, Hu et al. (2020) [12] found that HSR network promoted the migration of skill-intensive workers. Lin (2017) [29] also showed that industries with a higher reliance on nonroutine cognitive skills benefit more from HSR-induced market access to other cities. Theoretically, transportation infrastructure improvements can establish the economic links and traffic accessibility between different cities, broken the spatial barriers for economic activities, and expanded the market size. CVSC-TNS Research (CTR) report that people who take HSR in China are mainly business men and skilled workers with higher education attainment. Therefore, changes in traffic conditions and market environment induced by HSR are more likely to facilitate the flow of information and knowledge. Compared with labor-intensive firms, capital-intensive firm are more susceptible to these changes, and this study expects that the HSR stronger effects observed for capital-intensive firms. Based on the above analysis, we derive the following empirically testable hypothesis:

Hypothesis 3. Effects of HSR on firm-level resource misallocation exert firm-type heterogeneity and city-size heterogeneity.

## 3. Empirical Methodology

### 3.1 Measurement of firm-level resource misallocation

The firm-level resource misallocation are estimated following the Hsieh and Klenow (2009) [9] model of monopolistic competition with heterogeneous firms, which is based on the Melitz (2003) [30] closed economy model. The Hsieh and Klenow (2009) [9] model considers two types of distortions: output distortions (*τ*_*Ysi*_) and capital distortions (*τ*_*KSi*_). The profit function for firm *i* in sector *s* is given by:

$$\pi_{si}=(1-\tau_{Ysi})P_{si}Y_{si}-wL_{si}-(1+\tau_{Ksi})RK_{si}$$ (1)

where *P*_*si*_*Y*_*si*_ is the value added, *wL*_*si*_ is wage compensation, *R* is the rental price of capital, and *K*_*si*_ is the book value of fixed capital. Based on Hsieh and Klenow (2009) [9] calculation, the distortion parameters for output and capital are imputed as:

$$1-\tau_{Ysi}=\frac{\sigma }{\sigma -1}\frac{wL_{si}}{(1-\alpha_{s})P_{si}Y_{si}}$$ (2)

$$1+\tau_{Ksi}=\frac{\alpha_{s}}{1-\alpha_{s}}\frac{wL_{si}}{RK_{si}}$$ (3)

where σ is the elasticity of substitution between plants, and *α*_*s*_ is the share of capital in value added. The elasticity of substitution between plants is set to be equal to 3 as it is set in Hsieh and Klenow’s work. Hsieh and Klenow (2009) [9] motivated this choice by comparing literature on this very issue which provides estimates ranging from 3 to 10, and concluded that a conservative value of 3 should be chosen as the gains of liberalization are increasing in σ. For a robustness check, we also set a higher value of σ to be equal to 5. In the original Hsieh and Klenow (2009) [9] research, the rental price of capital (R) is set at 10%, which includes the real interest rate equals to 5% and a 5% depreciation rate. However, to make the rental rate more precise for China’s economy, we calibrate both components using relevant statistics and existing literature. According to the World Development Indicators database of the World Bank, the real interest rate in China during the period of 2009–2013 ranging from -1.40% to 5.53%. With a mean rate of 2.1%, we set real interest rate value at 2%. The depreciation rate is set at 6%, which is based on Zhang and Wang (2012) [31] estimation. As a result, the rental price of capital is equal to 8%. The share of capital *α*_*s*_ is set to be 1 minus the labor share in the corresponding sector in the United States. The US labor shares is used as the benchmark because the US economy is assumed to be comparatively less distorted. Using China’s actual labor compensation for labor share calculation would provide biased estimates of factor elasticity because distortions are potentially important in China. US labor shares are calculated based on the Annual Survey of Manufactures, which provides labor compensation and value added aggraded by sector. Consistent with Hsieh and Klenow (2009) [9] we multiply the obtained US labor shares by 1.5 to account for non-wage compensation. Firms that face output restrictions would have high output distortion *τ*_*Ysi*_, while firms that do not have access to credit would have high capital distortion *τ*_*Ksi*_.

The derivation of the productivity measure begins with a Cobb-Douglas production function. At firm level, Hsieh and Klenow (2009) [9] distinguished between physical productivity and revenue productivity. Physical productivity is computed using the following formula:

$${TFPQ}_{si}=A_{si}=k_{s}\frac{{\left(P_{si}Y_{si}\right)}^{\frac{\sigma }{1-\sigma }}}{K_{si}^{\alpha_{s}}{L_{si}}^{1-\alpha_{s}}}$$ (4)

where *k*_*s*_, which is not observed, is a scalar. Hsieh and Klenow showed that setting *k*_*s*_ = 1 does not affect relative productivities. Revenue productivity can be computed as:

$${TFPR}_{si}={P_{si}A}_{si}=\frac{{P_{si}Y_{si}}^{}}{K_{si}^{\alpha_{s}}{L_{si}}^{1-\alpha_{s}}}={\left(\frac{R}{\alpha_{s}}\right)}^{\alpha_{s}}{\left(\frac{1}{1-\alpha_{s}}\right)}^{1-\alpha_{s}}\frac{{\left(1+\tau_{ksi}\right)}^{\alpha_{s}}}{1-\tau_{Ysi}}$$ (5)

Eq (5) shows that TFPR does not vary across firms within the same industry if no distortion exists. Therefore, in the absence of distortions, more capital and labor would be allocated to firms with higher TFPQ, resulting in the exact same TFPR as firms with lower TFPQ [32] 单击或点击此处输入文字。.

Once firm-level TFPR are obtained, we can express average sectoral TFPR as:

$${\bar{TFPR}}_{s}=\frac{\sigma }{\sigma -1}{\left[\frac{R}{\alpha_{s}\sum_{i=1}^{M_{s}}\left(\frac{1-\tau_{Ysi}}{1+\tau_{Ksi}})(\frac{P_{si}Y_{si}}{P_{s}Y_{s}}\right)}\right]}^{\alpha_{s}}{\left[\frac{1}{(1-\alpha_{s})\sum_{i=1}^{M_{s}}\left(1-\tau_{Ysi})(\frac{P_{si}Y_{si}}{P_{s}Y_{s}}\right)}\right]}^{1-\alpha_{s}}$$ (6)

where *M*_*s*_ is the number of firms in sector *S*. If distortions were eliminated, and all the *TFPR*_*s*_ were equalized so that there were no more deviations from the mean, the sectoral TFP equals the average product productivity [33] 单击或点击此处输入文字。:

$${TFP}_{s}^{e}=\bar{A_{s}}={\left(\sum_{i=1}^{M_{s}}A_{si}^{\sigma -1}\right)}^{\frac{1}{\sigma -1}}$$ (7)

In this study, misallocation measures include both dispersion in TFPQ $\left(log\;({TFPQ}_{si}M_{s}^{\frac{1}{\sigma -1}}/\bar{A_{s}}),\right)$ and dispersion in TFPR $\left(log\;({TFPR}_{si}/\bar{{TFPR}_{s}})\right)$. Dispersion in TFPQ represents the distribution of firm-level total factor productivities (TFP). Any dispersion in TFPR represents a failure of allocative efficiency [10] 单击或点击此处输入文字。. Both distribution of TFPQ and TFPR can be affected by transportation improvement.

### 3.2 Baseline specification

In this study, the opening of HSR service is regarded as a “natural experiment”. Considering firms affected by HSR as the treated group, and those not affected as the control group, a DID model is employed. Conventional DID analysis assumes independence in data or no clustering effects. However, firm-level data in this study–yearly observations nested in firms and cities–have a hierarchical nature. Such a hierarchical structure can violate the assumption of independence in data required by the conventional DID analysis because firms located in the same city may not be independent [34] 单击或点击此处输入文字。. Within cities, firms share similar resources such as the institutional environment, accessibility, and the macroeconomic framework. Considering the structure of our dataset–a hierarchical structure with a balanced panel of multiple years of data on firms nested within cities, a 3-level multilevel model is employed to estimate the effect of HSR on resource misallocation among manufacturing firms to reduce the bias resulting from the nested structure. With the advantage of the multilevel model which does not require the independence in the errors and takes the interaction among the levels into account, the DID idea is incorporated and a multilevel DID model is finally applied. Following Hitzschke (2015) [35], we use the term “firms” *i* for level-1 units, “cities” *j* for level-2 units, and “years” *t* for level-3 units. The 3-level multilevel model is formally described in Eq (8):

$$Y_{ijt}=\gamma_{0}+\varphi X_{ijt}+\theta_{}{HSR}_{j}*{After}_{jt}+\beta Z_{jt}+\gamma_{}D_{t}+\vartheta_{t}+\delta_{jt}+\varepsilon_{ijt}$$ (8)

where *Y*_*ijt*_ represents misallocation measures, including dispersions in TFPQ and TFPR. *X*_*ijt*_ stands for a set of firm-specific characteristics, including firm age, firm size (number of employees), and firm ownership. *HSR*_*j*_ equals 1 if city *j* is connected to the HSR network. *After*_*jt*_ switches from 0 to 1 after the HSR started to operate in city *j* year *t*. The interaction term of *HSR*_*j*_ and *After*_*jt*_ captures the treatment effect of HSR. *Z*_*j*_ represents city-specific attributes, including city GDP per capita, and city population. *D*_*t*_ is the year dummies. *ε*_*ijt*_ is the level-1 residual, which is assumed to be identically independently distributed: $\varepsilon_{ijt}∼N(0,\sigma_{\varepsilon }^{2})$. *δ*_*jt*_ denotes the level-2 random term satisfying $\delta_{jt}∼N(0,\sigma_{\delta }^{2})$, and *ϑ*_*j*_ denoted the level-3 random term satisfying $\vartheta_{t}∼N(0,\sigma_{\vartheta }^{2})$. In the model, the only coefficients allowed to vary randomly are the random intercepts *δ*_*jt*_ and *ϑ*_*t*_, which means that the outcome variables are allowed to vary randomly across firms and cities.

The validity of the multilevel analysis bases on the assumption that the HSR stations were randomly assigned. However, in practice, China’s HSR project is aimed to connect provincial capital cities and those districts/counties with urban population larger than 500,000 (China State Council 2016), indicating that larger and richer cities are more likely to connect to the HSR network. To address the potential endogeneity, a two-stage estimation strategy is employed. The first-stage probability selection model is carried out to analyze the HSR stations placement problem using exogenous instruments and other city-level variables from the primary multilevel model. Following Duflo and Pande (2007) [36] and Diao (2018) [27], the city’s average elevation, average gradient, and historical railway passenger capacity of 1985 are applied as instruments in the first-stage estimation. The validity of the elevation, gradient, and historical railway passenger capacity as instruments is that they are closely correlated with the likelihood that a city is selected to build a HSR station but unlikely to affect the firm-level misallocation. The predicted values are then plugged back into the primary model, as the second stage [37] 单击或点击此处输入文字。.

### 3.3 Plausible channel–market potential

Studies that calculate market potential based on geodesic distances did not take the real transport network into account. The cost to reach locations is not determined by the straight-line distance, but the real transport network. Furthermore, as geodesic distances do not change over time, market potential calculated on the basis of geodesic distances could not reflect the impact of improved transportation infrastructure. To overcome these issues, our railway induced market potential change is calculated based on the real railway network. Market potential for each city is calculated as:

$${MP}_{jt}={pop}_{j}+\sum_{k}^{m}\frac{{pop}_{j}}{t_{jkt}}$$ (9)

*MP*_*jt*_ represents market potential of city *j* at time *t*, which measured the sum of own-city market size and the market size of other cities discounted by travel time. *t*_*jkt*_ is the shortest travel time (measured in minutes) along real railway network between city *j* and city *k* at time *t*. The change of shortest travel time between cities calculated on the basis of real railway network reflects the time-saving effect of HSR. The average population between 2009 and 2013 for city *j*, *pop*_*j*_, is applied in Eq (9) to eliminate the effect of population change on market potential. The constructed market potential index is similar to Holl (2012) [38].

City-level shortest travel time is derived based on the station-level shortest travel time applying the national train schedules of China. We then construct market potential index to quantify improvements of market potential induced by HSR construction. After determining the market potential changes, we proceed to explore the effects of HSR on resource misallocation resulting from the market potential change by incorporating interactive term between HSR treatment and market potential index to our mixed multilevel model. The multilevel model is specified as follows:

$$Y_{tij}=\gamma_{0}+\varphi X_{ijt}+\theta_{1}{MP}_{jt}+\theta_{2}{HSR}_{j}*{After}_{jt}+\theta_{3}{HSR}_{j}*{After}_{jt}*{MP}_{jt}+\beta Z_{jt}+\gamma_{}D_{t}+\vartheta_{t}+\delta_{jt}+\varepsilon_{ijt}$$ (10)

The estimated coefficient for the interactive term “*HSR*_j_*After_jt_*MP_*jt*_” will shed light on the underlying mechanism that HSR affects misallocation through market potential change.

### 4. Data sources

The empirical analysis is based on three main sources of data. First, the passenger rail travel time data used to calculate the shortest travel time between cities are collected from the official railway timetable available at the Chinese railway’s website (www.12306.com) by employing a web scraping technology. Previous research on the market potential measure usually computed based on geodesic distance [39, 40] 单击或点击此处输入文字。. However, geodesic distances do not vary over time, indicating that geodesic distance-based market potential measure could not reflect the evolution of railway network. To account the reduced travel time induced by HSR construction, the national train schedules are used to obtain the real railway travel information, including the arrival and departure time of all trains at each station, and the travel time between each station pair. Seven series of passenger train service are offered in China [16] 单击或点击此处输入文字。. The maximum speeds for each series of train service are shown in Table 1. While there is no single standard that applies worldwide, new lines in excess of 250 kilometers per hour (160 mph) and existing lines in excess of 200 kilometers per hour (120 mph) are widely considered to be high-speed. Based on the definition of HSR, three types of train services in China could be classified as HSR: the D-series trains, the G-series trains, and the C-series trains. In our dataset, there are 2071 D-series trains serving 169 cities, and 2410 G- and 1043 C-series trains serving 160 and 40 cities, respectively. To calculate market potential for each city, some assumptions need to be made to the data processing procedure, following [6] 单击或点击此处输入文字。. First, we assume that the change of travel time between cities is exclusively due to the opening of HSR services during the study period. Second, the shortest travel time of the conventional passenger rail services, including the K-, T-, and Z-series train services, is used to measure the travel time between cities before the introduction of the HSR service. Since the first HSR line in 2008 (Beijing-Tianjin line), China’s HSR network has expanded at a high speed. Shaw et al. (2014) [17] indicated that between 2009 and 2013, there was a boost of new HSR lines and reduction of ticket fares. To match the HSR expansion period and our research objective, we use the firm-level information between the year 2009 and 2013.

**Table 1:** Seven types of passenger train service.

Train Service Type	G	C	D	Z	T	K	P
Description	HSR Train	Inter-city HSR	CRH Train	Direct Express Train	Express Train	Fast Train	General Train
Maximum Speed (km/h)	[300, 350]	300	[200, 250]	160	140	120	100

Note: CRH train denotes the China Railway High-speed.

Data used to construct measures of firm-level resource misallocation are obtained from the Annual Survey of Industrial Firms (ASIF) for the period of 2009–2013 conducted by the National Bureau of Statistics of China. All the state-owned enterprises and non-state owned enterprises with annual sales of more than 5 million CNY in the manufacturing sector are surveyed. The information of the ASIF includes the firm’s industry (at the two-digit level), age (based on reported birth year), ownership, number of employees, wage payments, value-added, and capital stock. In the ASIF dataset, only wage payments are reported, and information on nonwage compensation is unknown. The median labor share in value added is roughly 35% in our dataset, which is lower than the aggregate labor share of 47% in manufacturing reported in the Chinese input-output table for 2012. We therefore assume that nonwage benefits are a constant share of a firm’s wage compensation, and calculate an adjustment factor such that the sum of imputed non-wage payments and wages equals 47% of aggregate value added. The annual value added of each firm is summed up its labor compensation (wage plus benefits), capital depreciation, operating profit, and taxes [41] 单击或点击此处输入文字。. For the ownership status, firms are classified into two categories: state-owned firms and non-state owned firms. The capital stock is defined as the book value of fixed capital net of depreciation. In this study, we focus only on manufacturing firms. To apply the US labor share during the calculation of misallocation, we drop the sectors that do not have close counterparts in the US industry. Due to entry and exit of firms, the ASIF provides an unbalanced panel, with roughly 300,000 firms reported each year. After data cleaning, there are totally 516,299 observations in our sample.

The city-level variables are obtained from China City Statistical Yearbook, 2009–2013. Data for 2010 is dropped due to missing observations. This dataset provides information on the number of population, and GDP per capita for prefecture-level cities. After dropping cities of missing data, there are 243 prefecture-level cities in our sample, 97 of them are connected to the HSR network and 146 of them had no HSR service during the study period. The summary statistics for the main variables are shown in Table 2. The column “Diff.” indicates the mean difference between sample with and without HSR service. From firm-level characteristics, firms located in cities with HSR service have more employees, higher wage compensation bills, and higher value added than those located in non-HSR cities. However, capital stock for firms in HSR cities are lower than those in non- HSR cities. One possible explanation is that firms located in HSR cities are younger, and therefore have lower depreciation rate and less intermediate inputs. Note that the “Diff.” descriptive statistics could be very different from the causal effect of HSR on firm performance, and t-tests may capture both the impact of HSR and the trends regarding cohort heterogeneity.

**Table 2:** Summary statistics.

Variable	Description	All sample		HSR = 0		HSR = 1		
		Mean	Std. Dev.	Mean	Std. Dev.	Mean	Std. Dev.	Diff.
***Firm Characteristics***								
Size	Number of employee	407.2	1230	377.0	989.3	419.4	1315	42.42*** (3.796)
Age	The age of the firm	10.75	7.588	10.94	7.831	10.27	7.477	-0.671*** (0.023)
State_owned	= 1 if state-owned	0.010	0.099	0.011	0.102	0.009	0.098	0.002**
								(0.003)
Value_Added (Mn CNY)	Nominal terms of value added	107.3	912.5	99.13	726.5	110.7	977.8	11.57*** (2.802)
Wage (Mn CNY)	Wage compensation (wage + benefits)	45.61	371.0	39.95	305.3	47.91	394.5	7.959*** (1.140)
Capital (Mn CNY)	Capital stock	58.85	573.5	61.68	553.2	57.70	581.5	-3.982** (1.761)
Obs.	Number of firms	516,299		148,974		367,325		
***City characteristics***								
Mkt_potential	Market potential	208.1	204.7	136.5	73.77	312.3	277.1	175.8*** (11.02)
Population (10,000 Person)	City population	152.2	188.1	99.77	66.43	228.2	265.7	128.4*** (10.52)
GDP (1000 CNY)	GDP per capita	54.59	36.77	47.58	32.23	64.81	40.45	17.23*** (2.139)
Obs.	Number of cities	243		146		97		

Note: The column “Diff.” gives the mean difference between sample with HSR service and sample without HSR service. Standard errors are in parentheses.

* p<0.10

**p<0.05

***p<0.01

## 5. Results and discussion

### 5.1 Market potential

**Fig 1:**
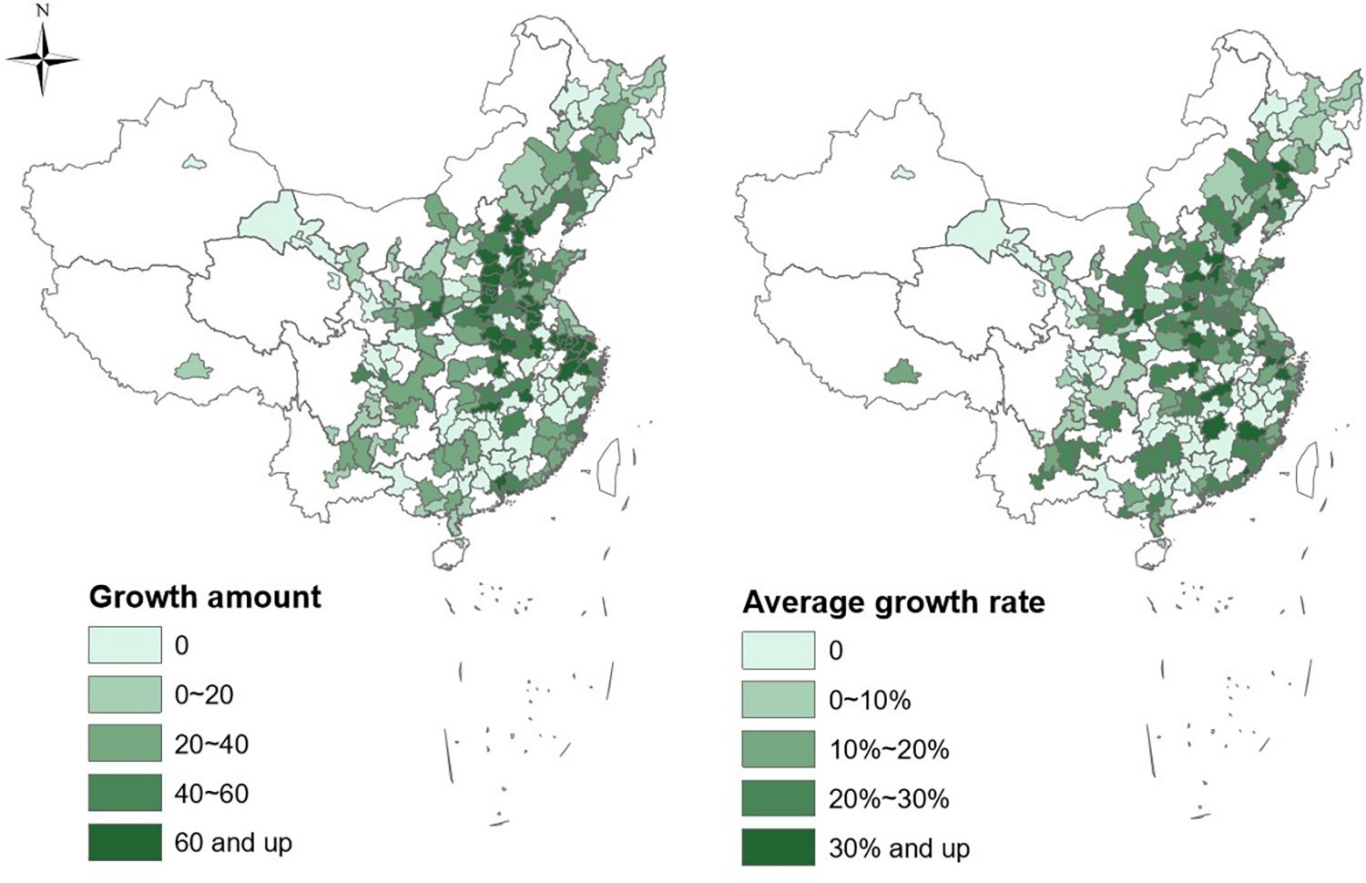
Changes of market potential for prefecture-level cities from 2009 to 2013.

In this study, we calculate the market potential for 243 prefecture-level cities based on real railway network for each year during 2009–2013. Fig 1 describes the growth amount and average growth rate of the market potential from 2009 to 2013, which provides a general idea about the change of a city’s reachable opportunities and resources. The blank areas of the figure indicate cities without statistic data or with no railway station. Generally, market potential of most cities increased due to the construction of HSR. The change amount and change rate surface of market potential shows the pattern of China’s railway network improvement. As a result of increasing HSR lines opening in the central east region during the study period, cities in this region gain more market potential increase than those in other regions.

Table 3 presents the means of market potential in 2009 and 2013 at different levels of spatial aggregation. The national mean of market potential is found increased by 10.94% from 196.6 in 2009 to 218.1 in 2013. Based on the city classification of China Business News (CBN) Weekly, we divide all the cities into 3 categories: big cities, medium cities, and small cities. Big cities include the first-tier cities in the CBN Weekly classification while medium cities include the second- and third-tier cities. The forth-tier cities are denoted as small cities. Though big and medium cities have higher level of market potential than small cities, small cities experienced higher market potential growth during 2009–2013. This indicates that small cities benefit more from the construction of HSR network. In China, smaller cities usually have less train stops with lower speed resulting in lower market potential. With lower baseline market potential, small decreases in travel time between small cities and other cities in the country would induce high growth rate.

**Table 3:** Summary statistics on market potential.

	Number of cities	Market potential (2009)	Market potential (2013)	Change (%)
Nation	243	196.6 (198.9)	218.1 (207.1)	10.94
Big Cities	19	642.6 (421.8)	698.6 (418.4)	8.715
Medium Cities	88	208.6 (108.9)	230.7 (113.0)	10.59
Small Cities	136	123.7 (59.32)	137.9 (66.74)	11.48

### 5.2 Dispersion of productivity

Before proceeding to the empirical estimation results, we would like to first describe the obtained results of the Hsieh and Klenow (2009) [9] framework. For both TFPQ and TFPR, we are interested in the relative to the mean distribution. Table 4 shows several measures of dispersion of TFPQ: the standard deviation, the 75^th^ minus the 25^th^ percentiles, and the 90^th^ minus the 10^th^ percentiles. Comparing the TFPQ distribution for firms located in HSR cities with those located in non-HSR cities, we find that firms located in HSR cities show larger TFPQ dispersion in the early stage of the HSR service. However, with the expansion of HSR service between 2009 and 2013, productivity of least productive firms located in HSR cities converged to the median TFPQ, and TFPQ dispersion of HSR firms became smaller than the dispersion of non-HSR firms in 2013. Table 4 also shows that TFPR experience more output distortions, while those to the right face higher capital distortions [33] 单击或点击此处输入文字。. The higher dispersion in 2013 indicates a worsening of resource allocation during the study period. Though variation of TFPR widened for all firms, firms located in HSR cities increased less than those located in non-HSR cities.

**Table 4:** Dispersion of productivity.

	2009		2013		Change(%)	
	HSR = 1	HSR = 0	HSR = 1	HSR = 0	HSR = 1	HSR = 0
***Dispersion of TFPQ***						
Std. Dev.	1.218	1.193	1.074	1.151	-11.82	-3.521
75 − 25	1.683	1.609	1.456	1.581	-13.49	-1.740
90 − 10	3.235	3.161	2.800	3.049	-13.45	-3.543
***Dispersion of TFPR***						
Std. Dev.	0.621	0.605	0.630	0.650	1.449	7.438
75 − 25	0.818	0.783	0.839	0.869	2.567	10.98
90 − 10	1.596	1.541	1.609	1.684	0.814	9.280

Note: Statistics are for deviations of log scaled TFPQ and log scaled TFPR from their industry means, respectively. Std. Dev. is standard deviation. 75 − 25 is the difference between the 75th and 25th percentiles, and 90 − 10 is the difference between the 90th and 10th percentiles. Industries are weighted by their value-added shares.

### 5.3 Empirical results

#### 5.3.1 Baseline estimates

**Fig 2:**
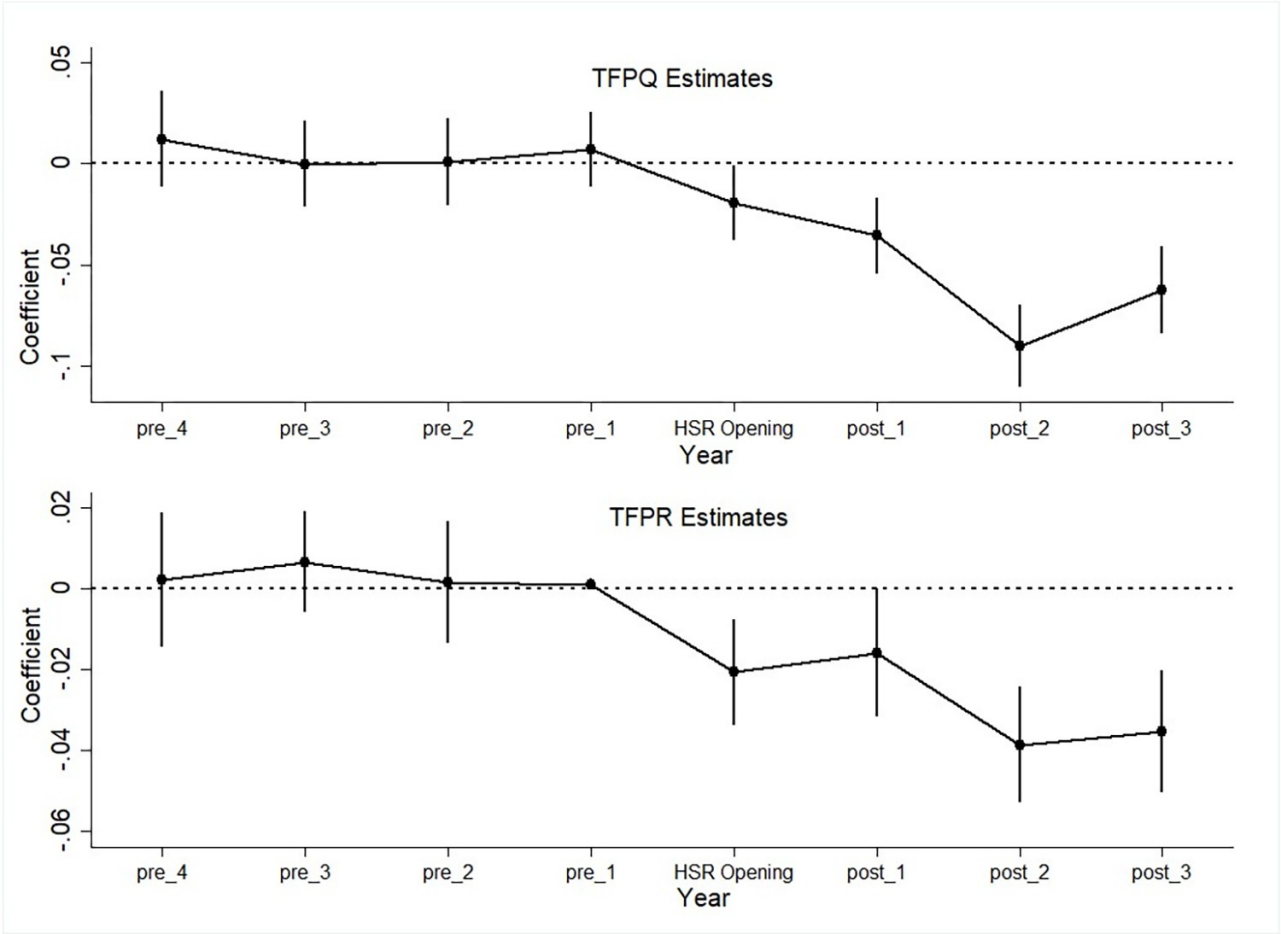
Pre-trend Test of TFPQ and TFPR Dispersions before and after the HSR Opening. Note: “pre_*” represents indicators of “*” year(s) before HSR opening, while “post_*” represents indicators of “*” year(s) after HSR opening. For each coefficient, the 95% confidence interval is reported.

This study aims to explore to what extent the HSR affects TFP distribution and resource allocation efficiency among manufacturing firms in China. However, a prerequisite for the validity of the empirical strategy is that the pre-trend of outcome variables between HSR firms and non-HSR firms should be similar. In this subsection, we generate eight year dummies both before and after the HSR’s opening, and plot the estimated coefficients with 95% confidence intervals in Fig 2. The coefficients of the year indicators represent the time path of the TFPQ and TFPR dispersions. All two graphs support the validity of the design since neither of the coefficients are significantly different from zero prior to the opening of HSR, indicating little difference in prior growth trend between the HSR firms and non-HSR firms. The graphs show a drop in TFPQ and TFPR dispersions right after the opening of HSR, and the largest effect is estimated to be seen two years after the HSR opening.

Following the model specification, we employ a 3-level multilevel model to access the effect of HSR on resource misallocation at firm level. To control for the non-random placement of HSR stations, we employ an IV strategy based on the city’s average elevation, average slope, and historical railway passenger capacity of 1985. In the first stage of the IV regression, we calibrate a logit model, utilizing city’s average elevation, average slope, and historical railway passenger capacity of 1985 and a set of city level characteristics (GDP per capita and population) to predict the probability that a city was connected to the HSR network during 2009–2013. The estimation result of the logit model is presented in Table 5. All instrumental variables are statistically significant, which suggests that city’s average elevation, average slope, and historical railway passenger capacity are good predictors of being a HSR city between 2009 and 2013, conditional on control variables. The predicted probability is then integrated in the second stage of the multilevel specification.

**Table 5:** Logit regression on the probability of being HSR cities in 2009–2013.

	Coef.	Std. err.
Slope	-0.217***	0.028
Elevation	-0.016***	0.000
1985 railway passenger capacity	0.045***	0.001
GDP	0.354***	0.002
Population	0.137***	0.001
LR Chi2(5)	40559.44	
Pseudo R2	0.167	

Note:

***Statistically significant at the 1% level

** 5% level

* 10% level.

Prior to the empirical analysis, the Intra-class Correlation Coefficients (ICC), which is equivalent to a random effect ANOVA [42] 单击或点击此处输入文字。, is calculated to determine whether a multilevel model is required to quantify the grouping effects. The value of the ICC reflects the ratio of between-group variance to the total variance. If the average productivity and resource misallocation is independent between firms and cities, the value of ICC tends to be zero, indicating that there is no between-group difference. In this case, the multilevel model is not required in the analysis. Applying the maximum likelihood method, the null model assessment results are presented in Table 6. The results indicate significant variation at levels 2 and 3 for both TFPQ and TFPR dispersions. Taking the null model result for $log\;(A_{si}M_{s}^{\frac{1}{\sigma -1}}/\bar{A_{s}})$, as an example, the calculated ICCs suggest that 10.49% of the total variation in physical productivity is located at the city level and 19.79% at the time level. In other words, 30.28% of the total variance in the sample is not located at the firm level. The results confirm the needs to use multilevel model to capture the intra-class effects.

**Table 6:** Fit statistics of the null model results.

	(1)	(2)
	TFPQ dispersion	TFPR dispersion
$\sigma_{\delta }^{2}$	0.204	0.492
$\sigma_{\vartheta }^{2}$	0.385	0.221
$\sigma_{\varepsilon }^{2}$	1.356	0.752
${ICC}_{\delta }=\frac{\sigma_{\delta }^{2}}{\sigma_{\delta }^{2}+\sigma_{\vartheta }^{2}+\sigma_{\varepsilon }^{2}}$	10.49%	33.79%
${ICC}_{\vartheta }=\frac{\sigma_{\vartheta }^{2}}{\sigma_{\delta }^{2}+\sigma_{\vartheta }^{2}+\sigma_{\varepsilon }^{2}}$	19.79%	15.08%

Table 7 presents the multilevel analysis results of regressing firm-level TFPQ dispersion and TFPR dispersion on HSR opening and other firm- and city-specific variables. The significantly negative coefficients of “HSR*After” in Model (1) and Model (2) indicate that the opening of HSR service decreased the disparity of TFP among manufacturing firms and increased allocative efficiency of production inputs. On average, the operation of HSR reduces the dispersion of TFPQ by 7.3%, while decreases the dispersion of TFPR by 8.9%. These findings corroborate the evidence in the literature that transportation improvement increases productivity and alleviates resource misallocation [1, 7] 单击或点击此处输入文字。. Increases in connectivity induced by HSR enable firms to reach distant markets faster and at lower costs, resulting in market expansions and increased levels of competition. A heightened level of competition raised the pressure on low productivity firms to increase their productivity level to survive in the market, otherwise they will be driven out of the market. The exit of low productivity firms and the increased productivity of these firms consequently reduced the dispersion of TFP. Reductions in travel times and travel costs decreased the barriers of reallocation of production inputs, and facilitate labor and capital moving to more productive firms.

**Table 7:** Impact of HSR on misallocation of manufacturing firms.

	(1)	(2)	(3)	(4)
	TFPQ_D	TFPR_D	TFPQ_D	TFPR_D
***Fixed parts***				
HSR*After	-0.073***	-0.089***	-0.027***	-0.038*
	(0.037)	(0.016)	(0.055)	(0.032)
MP			-0.043***	-0.058***
			(0.014)	(0.008)
HSR*After*MP			-0.019***	-0.024**
			(0.011)	(0.006)
Size	-0.049***	-0.032***	-0.050***	-0.032***
	(0.001)	(0.001)	(0.001)	(0.001)
State_owned	0.075***	0.097***	0.078***	0.098***
	(0.014)	(0.009)	(0.014)	(0.009)
Age	-0.011***	-0.003	-0.013***	-0.002
	(0.003)	(0.002)	(0.003)	(0.002)
GDP	-0.070***	-0.014*	-0.065***	-0.013*
	(0.013)	(0.006)	(0.010)	(0.006)
Population	-0.071***	-0.043***	-0.059***	-0.043***
	(0.014)	(0.009)	(0.017)	(0.010)
***Random parts***				
$\sigma_{\delta }^{2}$	0.190***	0.133***	0.215***	0.167***
	(0.009)	(0.006)	(0.009)	(0.007)
$\sigma_{\vartheta }^{2}$	0.354***	0.278***	0.354***	0.278***
	(0.001)	(0.001)	(0.001)	(0.001)
$\sigma_{\varepsilon }^{2}$	0.449***	0.259***	0.449***	0.259***
	(0.003)	(0.002)	(0.002)	(0.004)
***N***	505,462	506,446	505,462	506,446

Note: “TFPQ_D” and “TFPR_D” indicate TFPQ dispersion and TFPR dispersion respectively. Standard errors in parentheses.

* p < 0.1

** p < 0.05

*** p < 0.01.

Most of the control variables are statistically significant and with expected signs. Firm-level control variables include firm size, firm ownership (state owned or non-state owned), and firm age. Results in Table 7 show that smaller and younger firms are associated with relatively higher productivity disparities and misallocations than bigger and older firms. One possible explanation is that small and younger firms could barely secure external financial support for investment. With financial constraint, resource allocation could not match with firms’ productivity level, leading to resource misallocation. The estimation results also indicated that state-owned firms exhibit higher TFPQ and TFPR dispersions. In China, state ownership is often associated with implicit government guarantees and monopolistic powers. Government provides state-owned firms with monopolistic powers through a variety of policies that can distort competition and lead to resource misallocation by crowding out efficient non-state firms and allowing inefficient state-owned firms to survive [43] 单击或点击此处输入文字。. City-specific variables include GDP per capita and population. The GDP per capita intends to control for the change in firm-level productivity and resource misallocation due to changes in the economic environment. We find that higher GDP per capita lowers total factor productivity disparity among firms and improves allocative efficiency of production inputs. Generally, firms located in cities with large population exhibit lower resource misallocation and productivity dispersion.

#### 5.3.2 Heterogeneity effect of HSR

Even though firms located in the same city face similar transportation infrastructure and economic environment, they have different individual characteristics, which would shape different impact of HSR on firm-level productivity distribution and resource misallocation. The manufacturing sector contains a diverse range of manufacturing industries that differ greatly in capital and labor-use intensity [15] 单击或点击此处输入文字。. Existing literature indicated that capital-intensive firms and labor-intensive firms have different responses to changes of transportation infrastructure [28, 29] 单击或点击此处输入文字。. Due to its high price of tickets, business men and skilled workers account for a larger proportion of the total HSR passenger than general workers, which speeds up the exchange of information and knowledge. Therefore, we believe HSR would generate larger effect on capital-intensive firms than labor-intensive firms. To test this heterogeneity, our sample is categorized into two groups: capital-intensive firms and labor-intensive firms. The multilevel estimation results in Table 8 show that HSR significantly reduces TFPQ and TFPR dispersions for both capital- and labor-intensive firms. Coefficients for capital-intensive firms are larger in magnitude compared to the results for labor-intensive firms and for the whole sample presented in Table 7, indicating that capital-intensive firms benefit more from HSR service. HSR decreases the TFPQ dispersion of capital- and labor-intensive firms by 8.5% and 6.1%, respectively, while increases the allocative efficiency of capital- and labor-intensive firms by 10.9% and 6.8%, respectively. This finding is consistent with Li et al.’s (2017) [44] and Zhang et al. ’ s (2018) [45] conclusion that capital-intensive firms are more sensitive to the opening of HSR.

**Table 8:** Impact of HSR on misallocation by firm type.

	Labor-intensive firms				Capital-intensive firms			
	(1)	(2)	(3)	(4)	(5)	(6)	(7)	(8)
	TFPQ_D	TFPR_D	TFPQ_D	TFPR_D	TFPQ_D	TFPR_D	TFPQ_D	TFPR_D
***Fixed parts***								
HSR*After	-0.061*	-0.068*	-0.022***	-0.029*	-0.085***	-0.109***	-0.037***	-0.044**
	(0.060)	(0.035)	(0.068)	(0.047)	(0.037)	(0.088)	(0.052)	(0.068)
MP			-0.029***	-0.034***			-0.056	-0.067***
			(0.054)	(0.011)			(0.057)	(0.012)
HSR*After*MP			-0.017***	-0.020***			-0.024***	-0.029**
			(0.012)	(0.008)			(0.011)	(0.012)
Size	-0.044***	-0.015***	-0.045***	-0.015***	-0.058***	-0.027***	-0.057***	-0.012***
	(0.001)	(0.001)	(0.001)	(0.001)	(0.001)	(0.003)	(0.001)	(0.001)
State_owned	0.082***	0.091***	0.095***	0.094***	0.069***	0.106***	0.070***	0.105***
	(0.023)	(0.015)	(0.023)	(0.015)	(0.019)	(0.019)	(0.019)	(0.013)
Age	-0.008***	-0.003	-0.011***	-0.002	-0.015***	-0.005***	-0.014***	-0.007***
	(0.005)	(0.003)	(0.005)	(0.003)	(0.004)	(0.010)	(0.004)	(0.003)
GDP	-0.074***	-0.016***	-0.079***	-0.010***	-0.064***	0.011	-0.041***	-0.024***
	(0.015)	(0.009)	(0.015)	(0.009)	(0.016)	(0.008)	(0.014)	(0.013)
Population	-0.066***	-0.053***	-0.017	-0.038***	-0.103***	-0.037***	-0.057**	-0.067***
	(0.020)	(0.012)	(0.056)	(0.014)	(0.017)	(0.011)	(0.059)	(0.014)
***Random parts***								
$\sigma_{\delta }^{2}$	0.215***	0.128***	0.242***	0.171***	0.1765***	0.127***	0.160***	0.150***
	(0.054)	(0.056)	(0.055)	(0.056)	(0.055)	(0.056)	(0.055)	(0.057)
$\sigma_{\vartheta }^{2}$	0.349***	0.268***	0.349***	0.268***	0.352***	0.280***	0.352***	0.280***
	(0.005)	(0.004)	(0.005)	(0.004)	(0.006)	(0.005)	(0.006)	(0.005)
$\sigma_{\varepsilon }^{2}$	0.440***	0.255***	0.439***	0.255***	0.450***	0.264***	0.450***	0.264***
	(0.002)	(0.003)	(0.002)	(0.003)	(0.002)	(0.002)	(0.002)	(0.003)
N	264,356	241,106	264,356	241,106	264,869	241,572	264,869	241,572

Note: “TFPQ_D” and “TFPR_D” indicate TFPQ dispersion and TFPR dispersion respectively. Standard errors in parentheses. * p < 0.1

** p < 0.05

*** p < 0.01.

In addition to industry-specific effects of HSR on TFPQ and TFPR dispersions, we also investigate whether the HSR effect varies with city size. Different from aircraft which is traditionally a point-to-point link between larger cities, HSR connects large urban centers while bypassing smaller and less developed cities. Though some research has argued that construction of the HSR network can even result in economic activities being drained away from small and less-developed cities and bring more benefits to large cities [6, 46] 单击或点击此处输入文字。^,^ Zheng and Kahn (2013) [26] found a smaller effect of HSR on the mega cities than that on the nearby secondary cities. To explore how the HSR effect varies with city size, we create three subsamples based on city size: firms located in big cities, firms located in medium cities, and firms located in small cities. Estimation results presented in Table 9 indicate that HSR significantly decreased TFPQ dispersion of firms in all sizes of cities, but firms grouped in small cities gain more TFPQ dispersion rectification than those grouped in big and medium cities. As shown in Table 3, small cities gain the most connectivity increase from HSR construction. With improved connectivity, firms are more likely to select the optimal combination of production inputs with lower cost from a larger factor market, which in turn improves productivity. In regards to TFPR dispersion, HSR still exerts bigger effect on small cities This result is consistent with Yin et al.’s (2015) [47] finding that HSR connectivity brings new opportunities to commute or conduct business in small cities, which promotes development of these cities.

**Table 9:** Impact of HSR on misallocation by city scale.

	Big cities				Medium cities				Small cities			
	(1)	(2)	(3)	(4)	(5)	(6)	(7)	(8)	(9)	(10)	(11)	(12)
	TFPQ_D	TFPR_D	TFPQ_D	TFPR_D	TFPQ_D	TFPR_D	TFPQ_D	TFPR_D	TFPQ_D	TFPR_D	TFPQ_D	TFPR_D
***Fixed part***												
HSR*After	-0.061***	-0.076***	-0.021***	-0.037***	-0.065***	-0.096**	0.034	0.041***	-0.081***	-0.114***	-0.035*	-0.045***
	(0.018)	(0.010)	(0.090)	(0.051)	(0.037)	(0.017)	(0.116)	(0.067)	(0.052)	(0.024)	(0.172)	(0.099)
MP			-0.037***	-0.058***			-0.041***	-0.027*			-0.049**	-0.066***
			(0.033)	(0.019)			(0.024)	(0.014)			(0.023)	(0.014)
HSR*After*MP			-0.016***	0.020**			-0.008	-0.022***			-0.021*	-0.026***
			(0.014)	(0.008)			(0.021)	(0.012)			(0.027)	(0.016)
Size	-0.055***	0.032***	-0.055***	0.032***	-0.052***	0.034***	-0.052***	0.032***	-0.040***	0.028***	-0.041***	0.030***
	(0.001)	(0.001)	(0.001)	(0.001)	(0.001)	(0.001)	(0.001)	(0.001)	(0.002)	(0.001)	(0.002)	(0.001)
State_owned	0.048***	0.075***	0.047***	0.075***	0.130***	0.111***	0.107***	0.111***	0.126***	0.115***	0.125***	0.116***
	(0.017)	(0.012)	(0.017)	(0.012)	(0.020)	(0.013)	(0.020)	(0.013)	(0.034)	(0.023)	(0.034)	(0.023)
Age	0.005	-0.006**	0.005	-0.006**	-0.012***	-0.000	-0.008***	-0.000	-0.025***	-0.009***	-0.025***	-0.010***
	(0.004)	(0.003)	(0.004)	(0.003)	(0.004)	(0.003)	(0.004)	(0.003)	(0.006)	(0.004)	(0.006)	(0.004)
GDP	-0.068***	-0.018**	-0.070***	-0.014*	-0.086***	-0.008	-0.064***	-0.008	-0.080***	-0.013**	-0.037***	-0.018
	(0.015)	(0.008)	(0.015)	(0.008)	(0.020)	(0.009)	(0.015)	(0.009)	(0.020)	(0.011)	(0.019)	(0.011)
Population	-0.088***	-0.055***	-0.081***	-0.054***	-0.064***	-0.043***	-0.071***	-0.047***	-0.000	0.034***	0.026	0.039***
	(0.019)	(0.011)	(0.019)	(0.011)	(0.017)	(0.011)	(0.021)	(0.012)	(0.034)	(0.022)	(0.036)	(0.022)
***Random parts***												
$\sigma_{\delta }^{2}$	0.199***	0.167***	0.80***	0.188******	0.181***	0.165***	0.248***	0.171***	0.174***	0.115***	0.169***	0.113***
	(0.150)	(0.148)	(0.153)	(0.151)	(0.099)	(0.099)	(1.000)	(0.099)	(0.076)	(0.076)	(0.075)	(0.076)
$\sigma_{\vartheta }^{2}$	0.355***	0.290***	0.356***	0.290***	0.351***	0.284***	0.351***	0.284***	0.340***	0.280***	0.340***	0.279***
	(0.006)	(0.005)	(0.006)	(0.005)	(0.006)	(0.005)	(0.006)	(0.005)	(0.006)	(0.005)	(0.006)	(0.005)
$\sigma_{\varepsilon }^{2}$	0.455***	0.255***	0.454***	0.254***	0.441***	0.254***	0.441***	0.254***	0.444***	0.267***	0.444***	0.267***
	(0.003)	(0.003)	(0.003)	(0.003)	(0.002)	(0.002)	(0.002)	(0.002)	(0.003)	(0.003)	(0.003)	(0.003)
N	174,384	174,722	174,384	174,722	179,439	179,280	179,439	179,280	151,639	152,439	151,639	152,439

Note: “TFPQ_D” and “TFPR_D” indicate TFPQ dispersion and TFPR dispersion respectively. Standard errors in parentheses

* p < 0.1

** p < 0.05

*** p < 0.01.

#### 5.3.3 Mechanism at work—the role of market potential

Having shown that transportation infrastructure matters to firms’ total factor productivity distribution and resource misallocation, we now turn to the underlying mechanism. HSR modifies time-distance geography, and thus alters spatial access to opportunities. Researchers have constructed accessibility index to capture the combined effects of modifying time geography and redistributing opportunities. With respect to the impact of HSR on resource misallocation among manufacturing firms, one of the most direct effect is the improvement of market potential, which facilitates flows of production inputs. Thus, this study incorporates interactive term between HSR treatment and market potential gains into the baseline multilevel model to access the potential mechanism by which transportation infrastructure affects firm level resource misallocation. The estimated coefficients for the interactive terms will shed lights on the underlying mechanism of market potential. Model (3) and model (4) in Table 7 shows that averagely improvement of market potential rectifies total factor productivity disparity among manufacturing firms in China and improves allocative efficiency of production inputs. The coefficients for interactive terms are negative and statistically significant. Thus, firms located in HSR cities with relative larger market potential gain exhibit lower TFPQ and TFPR dispersions. One percentage increase in market potential boosts the effect of HSR on TFP dispersion and resource misallocation by 1.9% and 2.4%, respectively. Our results are aligned with Holl (2012) [38] and Wan and Zhang’s (2018) [20] finding that market potential is an important way through which the HSR affects efficient allocation of production resources. Industry-specific estimates in Table 8 indicate that the capital-intensive firms gain more productivity dispersion reduction and allocative efficiency improvement from HSR-induced market potential gains than labor-intensive firms. This is consistent with Zou et al.’s (2019) [48] finding that HSR was mainly passenger transport and promoted volume and speed of capital mobility. Dividing the sample based on city size, our results show that market potential strengthens the marginal effect of HSR on TFPQ and TFPR dispersions for all sizes of cities. Relative magnitude of coefficients suggests that firms located in small HSR cities benefit more from market potential increase. Resource misallocation denotes a situation in which labor and capital are not appropriately allocated based on their marginal revenue. The construction and expansion of HSR network provides greater market potential increase for small cities. Therefore, firms located in small cities are more likely to share resources across larger geographical space, resulting in more efficient matching between firms and production inputs.

To further test the theoretical hypothesis that improvement of market potential can improve allocative efficiency of production inputs through reducing geographic labor market segmentation and realizing agglomeration economies, we use local entropy index and regional wage disparity to measure agglomeration and labor market segmentation, respectively. Model (1) and Model (2) in Table 10 indicate that greater market potential induced by HSR remarkably reduces labor market segmentation and raises agglomeration. Model (3)–(6) show that both the decrease of labor market segmentation and increase of agglomeration significantly reduces TFPQ and TFPR dispersions. Therefore, reducing labor market segmentation and enhancing agglomeration are two important ways through which market potential affects efficient allocation of production resources among manufacturing firms.

**Table 10:** Impact of market potential on agglomeration and labor market segmentation.

	(1)	(2)	(3)	(4)	(5)	(6)
	Agglomeration	Segmentation	TFPQ_D	TFPR_D	TFPQ_D	TFPR_D
HSR*After	0.186*** (0.006)	-0.369*** (0.006)	-0.039* (0.002)	-0.021** (0.001)	-0.038*** (0.002)	-0.020*** (0.001)
MP	0.070*** (0.002)	-0.112*** (0.002)	-0.018* (0.050)	-0.081** (0.003)	-0.020* (0.050)	-0.085** (0.003)
HSR*After*MP	0.034*** (0.001)	-0.065*** (0.001)	-0.008** (0.003)	-0.033** (0.002)	-0.008*** (0.003)	-0.031*** (0.002)
Agglomeration			-0.008*** (0.001)	-0.004*** (0.002)		
Segmentation					0.082*** (0.004)	0.061*** (0.002)
Controls	Yes	Yes	Yes	Yes	Yes	Yes
N	499,330	499,330	486,118	487,928	486,118	487,928

#### 5.3.4 Robustness checks

To have more confidence in the estimation results, additional robustness checks are conducted. First, to check the robustness of the obtained source misallocation results, we performed calculations of the productivity dispersion with another elasticity of substitution between plants value-added (σ = 5). Table A1 in Online Appendix of S1 Appendix shows that the TFPR dispersion increases while TFPQ dispersion decreases with respect to the value of elasticity of substitution between firms’ value added. Hsieh and Klenow (2009) [9] explain this property of their model as when σ is higher, TFPR gaps are closed more slowly in response to reallocation of inputs.

Additionally, in the empirical analysis above, we regard the opening of HSR as a natural experiment, and DID strategy is applied. Since the number of HSR trains in each city could also affect resource allocation among manufacturing firms, we replace the opening of HSR in Eq (10) with total number of HSR trains. Table A2 in Online Appendix of S1 Appendix shows the estimation results, which tests whether or not there are some differences in the impacts of the opening and service intensity of HSR on resource misallocation. We can see that every 1% increase in service trains cause TFPQ and TFPR dispersions to decrease by 9.7% and 6.8%, respectively. The mechanism estimation results are also consistent with those in the main empirical analysis that increase of the market potential boosts the marginal effect of HSR on TFPQ and TFPR dispersions. Furthermore, considering that there are several measures of resource misallocation, we replace TFPQ and TFPR dispersions in Eq (10) with capital distortion Log(1+*τ*_*Ksi*_) and output distortion Log(1-*τ*_*Ysi*_), respectively. The depend variable Log(1-*τ*_*Ysi*_) takes a negative value if firms are restricted from growing to their efficient size, while a positive value of Log(1+*τ*_*Ksi*_) indicates capital constraint relative to labor. The estimation results are reported in Table A3 in Online Appendix of S1 Appendix. We find that firms located in HSR cities experience lower output and capital distortions, and with 1% increase in market potential gain, capital and output distortion will decrease by 4.2% and 4%, respectively. This reflects that HSR promotes manufacturing firms growing to their efficient size and reduces their capital distortion. All these results discussed in this section appear to be robust in their magnitude and statistical significance, confirming the robustness of our main empirical results.

## 6. Conclusions

Since the opening of China’s first HSR—Beijing-Tianjin intercity line—in 2008, HSR has become an extremely important mode of transportation that affects China’s economic geography and accelerates the reallocation of resources. Given China’s highly developed HSR networks, this study used data for 2009–2013 to access the impacts of HSR on resource misallocation among manufacturing firms. Incorporating the DID idea into a multilevel model, this study comes out with the following main conclusions. As a whole, the opening of HSR in a city significantly rectifies resource misallocation among manufacturing firms. HSR services shorten space-time distances between cities and induce the reallocation of production resources from low-efficient firms to productive ones. Also, firms grouped in small and medium cities experience higher TFPQ and TFPR dispersion reductions than big cities. This implies that HSR facilitate the diffusion of resources from big cities to the surrounding small and medium cities more easily. However, it should be noted that although many cities may not directly connect to HSR network, they can still benefit from HSR services because travelers in non-HSR cities can access HSR through other transport modes (Diao, 2018). Due to this spillover effect of HSR services, our estimation may underestimate the impact of HSR on resource misallocation. In addition, when considering industry heterogeneity, our results show that the benefits from HSR are substantially more accrued to the capital-intensive firms. As business men and skilled workers account for a large proportion of the total HSR passenger, accessibility improvement induced by HSR promotes the flow of personnel, information, and knowledge. Therefore, capital-intensive firms are more sensitive to the opening of HSR. Underlying mechanism analysis shows that increase of the market potential boosts the marginal effect of HSR on TFPQ and TFPR dispersions through reducing labor market segmentation and increasing agglomeration, confirming that market potential gain is an important way through which the HSR affects productivity distribution and efficient allocation of production inputs among manufacturing firms in China. HSR improves accessibility to input and output markets, and thus increases market potential. The extent of market potential is an important determinant of firms’ productivity. Greater market potential allows firms enjoy larger labor market pool and input share, which leads to efficient allocation of resources among firms. The robustness checks indicate that our findings are robust and consistent under various settings.

The construction and expansion of HSR network is considered to be an important way of improving the efficiency of economic development. This study provides useful insights in understanding how HSR affects macro economic productivity through its impact on firm-level resource misallocation. Based on the findings of this study following policy implications are put forward. First, it is necessary to further develop and expand China’s HSR network. HSR has a significant role in improving the resource allocation efficiency at firm level. The planning and construction of HSR network promotes cross-regional flow of resources, which is crucial for productivity growth. Second, when improving transportation infrastructure, city heterogeneity should be taken into consider. Empirical studies on the HSR network have confirmed the agglomerative effect of the HSR. However, by significantly shortening the rail travel time among major Chinese cities, HSR facilitates firms or production activities to locate together to enjoy positive externalities, which may result in excessive concentration of capital and labor in big cities. Therefore, it is important to balance transportation investment among cities of different sizes. Furthermore, other infrastructure, such as communication infrastructure and energy infrastructure, should be improved in small cities. To fully take advantage of HSR network and ensure the sustainable productivity growth of firms in small cities, it is necessary to increase investment in other infrastructure in less developed cities. Finally, regional industrial distribution should be taken into account in the future HSR network planning. Compared with labor-intensive firms, HSR plays a more important role in promoting resource allocation and productivity improvement in capital-intensive firms. In view of this, in the future planning and construction of HSR network, more investment should be given to regions with a large proportion of capital-intensive firms.

## Supporting information

S1 Appendix (DOCX)
