# Supplementary material for: Unraveling the rectifying role of transportation improvement on resource misallocation among manufacturing firms in China–Facts and mechanism
Source: PLoS One. 2023 Aug 17;18(8):e0288390. doi: 10.1371/journal.pone.0288390 (PMC10434915; doi:10.1371/journal.pone.0288390)
Supplement: S1 Appendix — (DOCX) [file pone.0288390.s001.docx]

Online Appendix

**Table A1. Robustness check of productivity dispersion (****σ=5)**

|  | 2009 | | 2013 | | Change(%) | |
| --- | --- | --- | --- | --- | --- | --- |
|  | HSR=1 | HSR=0 | HSR=1 | HSR=0 | HSR=1 | HSR=0 |
| ***Dispersion of TFPQ*** |  |  |  |  |  |  |
| Std. Dev. | 1.090 | 1.074 | 0.847 | 0.899 | -22.29 | -16.29 |
| 75 − 25 | 1.513 | 1.422 | 1.152 | 1.237 | -23.86 | -13.01 |
| 90 − 10 | 2.856 | 2.789 | 2.175 | 2.340 | -23.84 | -16.09 |
| ***Dispersion of TFPR*** |  |  |  |  |  |  |
| Std. Dev. | 0.631 | 0.621 | 0.639 | 0.657 | 1.268 | 5.797 |
| 75 − 25 | 0.851 | 0.821 | 0.871 | 0.925 | 2.35. | 12.67 |
| 90 − 10 | 1.63 | 1.596 | 1.654 | 1.719 | 1.472 | 7.707 |

**Table A2. Impact of Service Intensity of HSR on Misallocation**

|  | (1) | (2) | (3) | (4) |
| --- | --- | --- | --- | --- |
|  | TFPQ_D | TFPR_D | TFPQ_D | TFPR_D |
| ***Fixed parts*** |  |  |  |  |
| Number of trains | -0.097^***^ | -0.068^***^ | -0.048^***^ | -0.038^***^ |
|  | (0.009) | (0.006) | (0.019) | (0.009) |
| MP |  |  | -0.022 | -0.012^***^ |
|  |  |  | (0.052) | (0.031) |
| Number of trains *MP |  |  | -0.021^***^ | -0.015^*^ |
|  |  |  | (0.026) | (0.016) |
| Size | -0.101^***^ | -0.029^***^ | -0.102^***^ | -0.031^***^ |
|  | (0.002) | (0.001) | (0.002) | (0.001) |
| State_owned | 0.094^***^ | 0.137^***^ | 0.068^**^ | 0.142^***^ |
|  | (0.032) | (0.021) | (0.033) | (0.019) |
| Age | -0.013 | -0.020^***^ | -0.030^**^ | -0.023^***^ |
|  | (0.011) | (0.007) | (0.013) | (0.005) |
| GDP | -0.050^*^ | -0.112^***^ | -0.116^**^ | -0.112^***^ |
|  | (0.047) | (0.031) | (0.054) | (0.019) |
| Population | -0.046^***^ | -0.031^***^ | -0.051^***^ | -0.033^***^ |
|  | (0.050) | (0.031) | (0.054) | (0.022) |
| ***Random parts*** |  |  |  |  |
| $\sigma_{\delta}^{2}$ | 0.304^***^ | 0.406^***^ | 0.239^***^ | 0.351^***^ |
|  | (0.027) | (0.037) | (0.020) | (0.029) |
| $\sigma_{\vartheta}^{2}$ | 0.569^***^ | 0.707^***^ | 0.550^***^ | 0.707^***^ |
|  | (0.002) | (0.003) | (0.002) | (0.003) |
| $\sigma_{\varepsilon}^{2}$ | 0.511^***^ | 0.898^***^ | 0.509^***^ | 0.898^***^ |
|  | (0.001) | (0.002) | (0.001) | (0.002) |
| ***N*** | 505,462 | 506,446 | 505,462 | 506,446 |

Note: “TFPQ_D” and “TFPR_D” indicate TFPQ dispersion and TFPR dispersion respectively. Standard errors in parentheses. * p < 0.1, ** p < 0.05, *** p < 0.01.

**Table A3. Impact of HSR on Output and Capital Distortions**

|  | (1) | (2) | (3) | (4) |
| --- | --- | --- | --- | --- |
|  | Log(1-$\tau_{Ysi}$) | Log(1+$\tau_{Ksi}$) | Log(1-$\tau_{Ysi}$) | Log(1+$\tau_{Ksi}$) |
| ***Fixed parts*** |  |  |  |  |
| HSR*After | -0.166^***^ | -0.136^**^ | -0.068^***^ | -0.060^***^ |
|  | (0.012) | (0.013) | (20.441) | (0.015) |
| MP |  |  | -0.042^***^ | -0.040^***^ |
|  |  |  | (0.001) | (0.003) |
| HSR*After*MP |  |  | -0.044^***^ | -0.034^***^ |
|  |  |  | (0.008) | (0.002) |
| Size | -0.093^***^ | -0.006^***^ | -0.163^***^ | -0.007^***^ |
|  | (0.017) | (0.000) | (0.025) | (0.000) |
| State_owned | -0.180 | -0.024^***^ | -1.259^**^ | -0.011^***^ |
|  | (0.329) | (0.004) | (0.518) | (0.003) |
| Age | -0.069^***^ | -0.004^***^ | -0.138^***^ | -0.002^**^ |
|  | (0.014) | (0.002) | (0.023) | (0.001) |
| GDP | -0.083^***^ | -0.052^**^ | -0.178^***^ | -0.065^***^ |
|  | (0.020) | (0.022) | (0.034) | (0.003) |
| Population | -0.047^**^ | -0.024 | -0.131^***^ | -0.108^***^ |
|  | (0.020) | (0.021) | (0.003) | (0.004) |
| ***Random parts*** |  |  |  |  |
| $\sigma_{\delta}^{2}$ | 3.517^***^ | 0.036^***^ | 5.942*** | 0.064*** |
|  | (0.285) | (0.003) | (0.472) | (0.006) |
| $\sigma_{\vartheta}^{2}$ | 3.451^***^ | 0.089^***^ | 3.455*** | 0.097*** |
|  | (0.016) | (0.001) | (0.016) | (0.001) |
| $\sigma_{\varepsilon}^{2}$ | 4.557^***^ | 0.090^***^ | 4.532*** | 0.090*** |
|  | (0.009) | (0.001) | (0.009) | (0.001) |
| ***N*** | 505,462 | 506,446 | 505,462 | 506,446 |

Note: “TFPQ_D” and “TFPR_D” indicate TFPQ dispersion and TFPR dispersion respectively. Standard errors in parentheses. * p < 0.1, ** p < 0.05, *** p < 0.01.
